# Health Care 2025: How Consumer-Facing Devices Change Health Management and Delivery

**DOI:** 10.2196/60766

**Published:** 2025-04-23

**Authors:** Simon Trinh, Devin Skoll, Leslie Ann Saxon

**Affiliations:** 1 Center for Body Computing University of Southern California Playa Vista, CA United States; 2 Keck School of Medicine University of Southern California Los Angeles, CA United States; 3 New York Presbyterian - Columbia University Irving Medical Center New York, NY United States

**Keywords:** decentralized, digital health, consumer, health care, COVID-19, wearables, medical devices, health management, mHealth, wearable, well-being, care delivery

## Abstract

Embarking on a journey into the future of health care shaped by technological advances and the impact of the COVID-19 pandemic, we delve into the transformative landscape shaped by the integration of wearable technology, medically regulated devices, and advanced software. The ability to offer consumers unprecedented access to vital signs, advanced biomarkers, and environmental data enables a host of new capabilities to fill gaps in existing knowledge and permit individualized insights and education. Continuous monitoring enables individualized insights, emphasizing the need for a redefinition of health and human performance that is decentralized, dynamic, and personalized. The challenge lies in managing the massive amounts of continuous wearable data, necessitating new definitions of health data and secure practices. The COVID-19 pandemic has accelerated the adoption of digitalized consumer-facing diagnostics and software, transforming the traditional patient role. Consumers now have the tools to identify and understand an impending or existing disease state before they encounter traditional health care delivery health systems, making self-diagnosis commonplace. This shift empowers consumers to actively participate in their health, contributing to a new era where patients are in control of their well-being, from wellness to disease. Physicians in 2025 will engage with more informed and educated consumers, leveraging advanced analytic tools for diagnostics and streamlined patient management. Wearable devices play a pivotal role in enhancing patient engagement, while virtual reality and tailored software can be used by physicians to offer immersive learning experiences about conditions or upcoming procedures. Clinician decision support models and virtual care solutions will contribute to recruiting and maintaining health care providers amid a growing workforce shortage. Health care delivery organizations are transforming to improve outcomes at a lower cost, with partnerships with digital technology companies enabling innovative care models. This marks a historic moment where digital health and human performance solutions empower consumers to actively participate in their care. Physicians embrace digital tools, fostering richer patient partnerships, while health care organizations seize unprecedented opportunities for multilocation care delivery, addressing cost, workforce, and outcome challenges.

## Introduction

After nearly 15 years of commercial availability, consumer-facing wearable devices are owned by one in three Americans and now have medical-grade diagnostic technology [1]. Since the COVID-19 pandemic especially, these devices have exploded in popularity, offering the average consumer basic information about their health and habits. Medically regulated devices like wearable continuous glucose monitors, which are Food and Drug Administration–approved for diabetic management, also have great potential to provide unique insights into metabolism for nondiabetics [2]. These technologies have arisen in response to unmet needs for remote, portable, and medically viable technology, and the pandemic accelerated the development of these solutions. This year, wearable devices and user-facing software will bring significant health and human performance data and insights to the public, all occurring outside of medical facilities. These advanced capabilities, now available for health and human performance needs, mirror a broad societal shift reflecting consumer expectations for digital access to personalized data across finance, entertainment, and other commerce [3]. These sectors have been transformed by digital innovation creating novel products and workflows. The consumer benefits include efficient and immediate access to information, products, and cost transparency. This paper explores how digital transformation driven by wearable consumer technology, medically regulated devices, and modern-day software both challenge and redefine the traditional role of the patient, physician, and health care delivery organization.

## What, How, and Where We Measure Blurs the Lines Between Health and Disease

**Figure 1:**
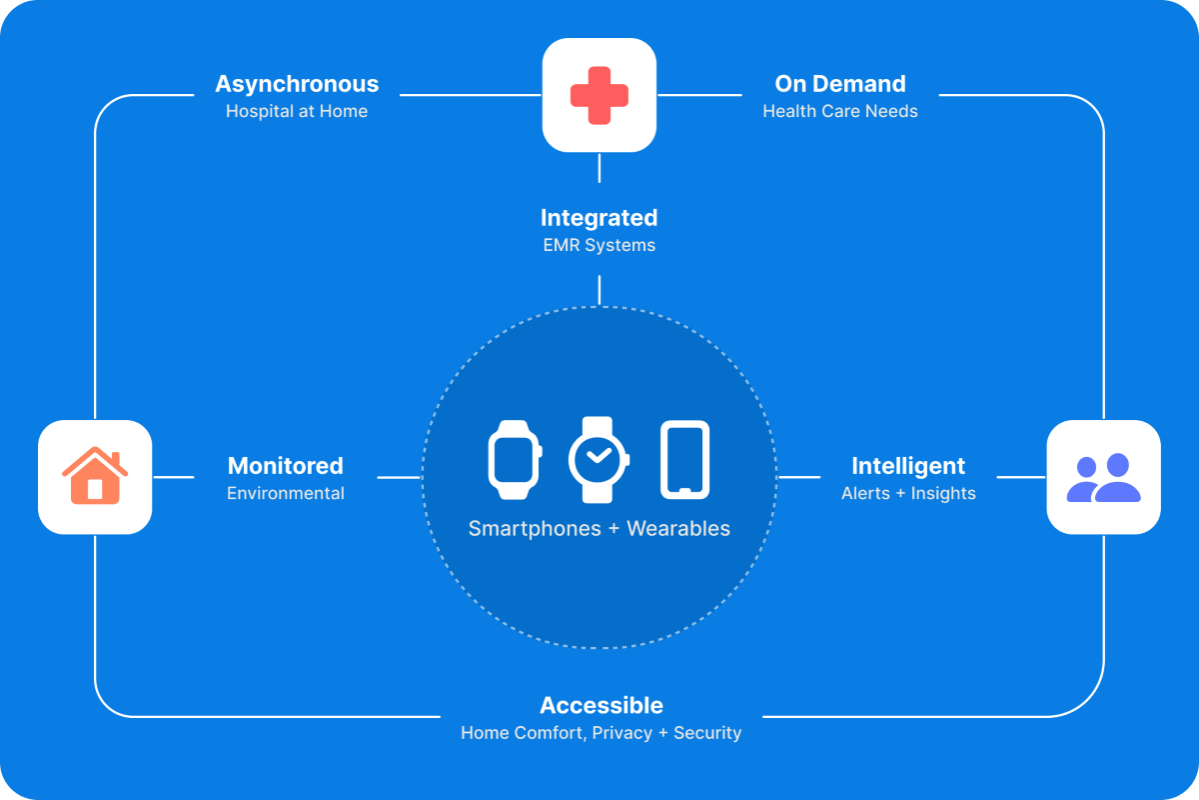
Interconnectivity of wearables and devices with the environment, health care systems, and individuals. EMR: electronic medical record.

In 2025, vital signs like heart rate and rhythm, blood pressure, body temperature, and oxygen saturation are available to consumers from a single wearable device [4]. Emerging technologies like vital signs from a face photo app on a smartphone are on the horizon, promising quicker, less invasive testing [5]. Compact devices embedded with activity tracking and sleep sensors are also capable of providing balance and fall information, continuous and peak oxygen consumption, sound exposure, and environmental safety tracking, enabling a host of new capabilities that can fill in gaps in existing knowledge. The ability to track the earliest risk of a disease state with a novel tool kit of digital biomarkers that establish baseline behavior and can measure improvement and degradation permits individualized insights [6]. Integration of ambient environmental data like temperature, air quality, and violence—all recognized to impact health—provide the opportunity to mitigate risk. In addition to continuous glucose sensing, continuous lactate and ketone sensing will provide data and insights into the relationship between nutritional content, activity, and recovery (Figure 1) [7].

**Figure 2:**
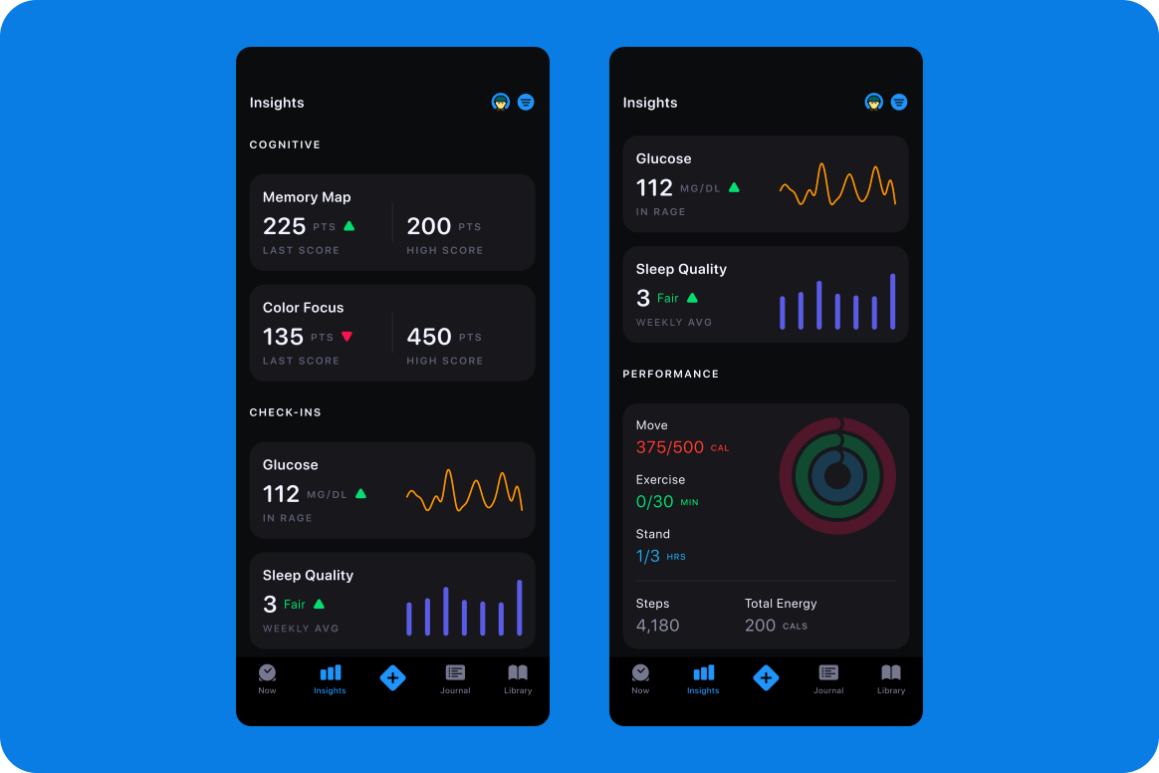
Sample measurements taken from the USC Center for Body Computing Lightning app. Results comprise physiologic data from wearables and sensors, alongside self-assessment and survey data.

The USC Center for Body Computing, for example, has developed a digital research software platform that is capable of continuous assessment of self-reported psychological state and can dynamically measure cognitive status [2]. These datasets, when integrated, provide measures of holistic health and help to drive a deeper understanding of their relatedness, in the moment or as a trend, providing each individual not only data but also insights into how to make the best health and human performance decisions (Figure 2). Because these tools are measuring the consumer continuously, the line between health and disease states blurs, requiring a redefinition of health and human performance that is much more individualized and dynamic. This “de-siloing” of health and disease presents significant challenges for a health care system built and defined largely by what happens inside a medical facility to patients who are sick. In the past few years, health care has begun to shift away from the hospital environment and into patients’ homes, leading to the rise of new paradigms to match this change.

Data collection, storage, integration, and analysis are some of the opportunities and challenges faced in provisioning new health and human performance care. Security in data, device and software design, and implementation all require new definitions of what constitutes health data and secure practices. Data existing in electronic medical records will seem limited compared to the massive amounts of continuous wearable data generated at the level of individuals. How to confidentially secure and interoperate that data to benefit discovery and care for the consumer as well as traditional and nontraditional health care provision will require serious work and the leveraging of new tools like large language models and machine learning, which are themselves incompletely understood [8].

## Digitalization, Health Care Consumerism, and the Patient

The word patient, a label provided to people who encounter brick-and-mortar health care facilities with or seeking a diagnosis, has a meaning deeply associated with an illness. The COVID-19 pandemic necessitated and accelerated use and understanding of the benefits of digitalized consumer-facing diagnostics and software. The spectrum of issues surrounding the pandemic expanded our understanding of the utility and scope of use of connected technology and consumer-facing diagnostics [9]. COVID-19 pandemic management went beyond detecting the presence of infection. Technology was used to track the progression to an infected status through serial testing, while the recovery period required testing, symptom management, and the requirement to continuously monitor health status for recurrence and infectivity.

In 2025 and beyond, consumers will have even more tools to diagnose, understand, and manage an impending or existing disease state before they encounter traditional health care delivery systems. When consumers eventually seek telehealth-enabled or in-person visits to health care systems, they will possess a novel type of digital health care literacy and will often arrive with a self-diagnosis. This new health care consumer evolves the traditional patient role, as they will be the ones providing data to the clinician and will expect partnership, ongoing communication, and ongoing bidirectional data sharing in their health and human performance care path [10]. Consumer access to large language learning models embedded in consumer software will also assist in elevating consumers’ medical knowledge.

**Figure 3:**
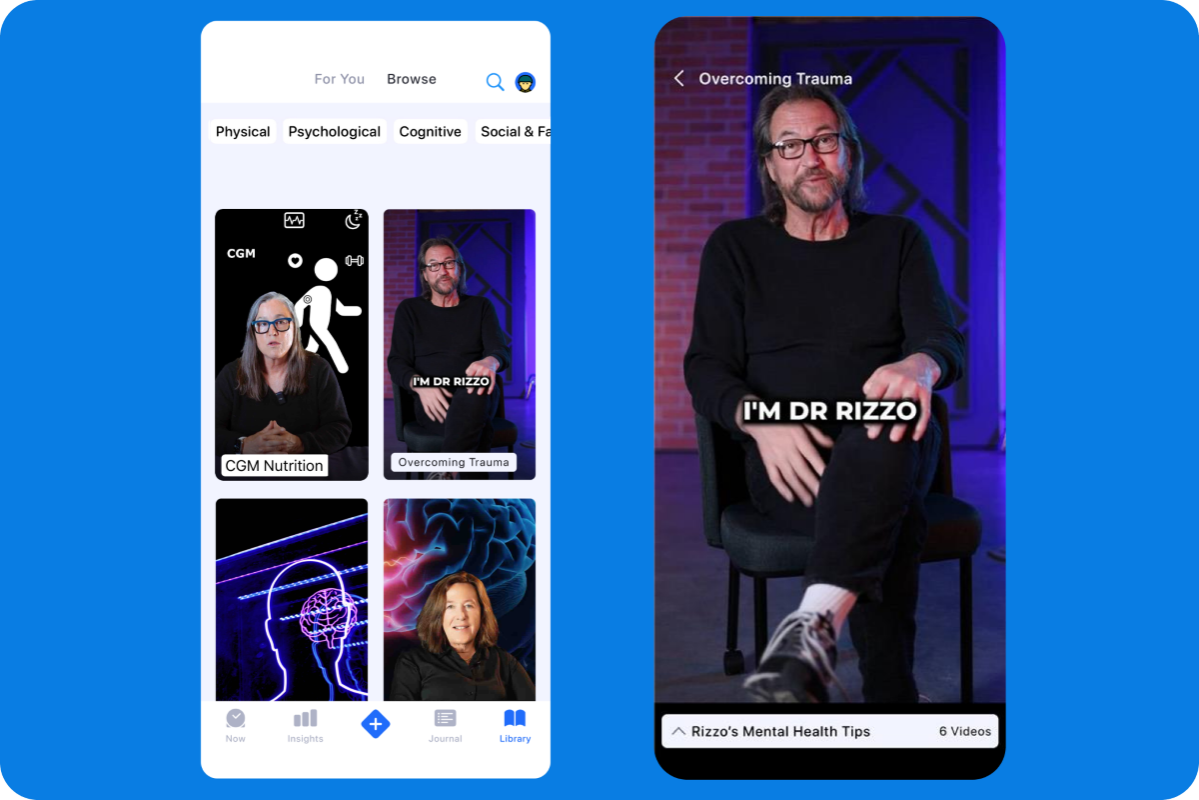
Use of short-form video content within the Center for Body Computing Lightning app to improve health literacy on general health improvement. On left, video library showcasing videos on nutrition and psychological health. On right, short-form video on mental health featuring a psychologist (all individuals have given consent for their likeness to be used).

The US health care system is fragmented and difficult to navigate, lacking accessible tools to improve health literacy. The future of digital health relies on educating patients throughout their lives using simple and engaging interfaces that scale to populations with connected devices. One of the greatest barriers to health literacy and health service delivery is navigating insurance and the paternalistic nature of health care, which forgoes patient autonomy for expedience. Digital health and the advent of artificial intelligence can help solve these issues by providing virtual assistants, chatbots, and educational sources that help patients understand their health and navigate the health care system. For example, our own digital health and human performance software provides the user immediate access to education surrounding a finding like hyperglycemia that is tailored toward explaining the concept of insulin resistance and its management, with short-form video content on a variety of subjects available on demand (Figure 3). Ultimately, these changes signal a new era of medicine—one where patients are more in control of their health, from wellness to disease, and physicians and health systems alike have novel opportunities to deliver patient-centered, value-based care because they have the insights that come from continuous monitoring and patient engagement [11].

## The Assisted Physician and Allied Care Professional

The physician or allied care professional practicing in 2025 will engage with a more informed and educated consumer, and they will have access to a variety of advanced intelligence and analytic tools to assist in diagnostics and streamlined patient management that integrates consumer-generated health data [12]. Wearable devices that help drive patient engagement and understanding present a wonderful opportunity for care providers to ensure that the information, education, and decision-making they provide to patients is retained and updated. Tools like virtual reality headsets and tailored software can be used to provide patients with immersive learning experiences about conditions, preexposing them to a procedure or treatment and even the postoperative course or side effects to better prepare them and their caregivers. Given the depleted and growing provider workforce shortage, novel tools and care models can also help recruit and maintain providers in the workforce. Physicians will not need to be physically tied to a medical workspace to provide virtual care solutions and can work in multidisciplinary teams to research and create “anywhere, anytime” models of care that offer increased flexibility to work schedules [13,14].

## Health Care Delivery Organization Transformation

Improving outcomes at a lower cost is a reality for health care delivery organizations in 2025. Significant partnerships with digital technology companies enable care models such as hospital-at-home for acute, subacute, and rehabilitation care. Early data from these programs indicates improved outcomes and cost savings for both acute and posthospitalization recovery programs when they are delivered in the home [15,16]. Additionally, these novel health care delivery models decompress hospitals’ acute and chronic care bed requirements and offer staff alternative work schedules and flows. Integrating consumer-collected digital wearable data can be used to drive a symptomatic or asymptomatic consumer to seek care for conditions that include cardiovascular, metabolic, musculoskeletal, infectious, and oncologic care at the earliest moment [17,18]. Preventative care, informed by engaged patients helping to drive their outcomes, can deliver value-based care that is highly dependent on patient behaviors and understanding outside of the confines of a medical center. Investment in confidential computing models, cybersecurity, and advanced analytics will identify further opportunities for evolving care and drive opportunities across the spectrum of primary care and beyond.

In 2025, traditional health care delivery organizations will also have a host of new companies from Best Buy to Amazon that will help provision and provide distributed care models or will compete for primary care visits. Amazon is offering its 200 million Amazon Prime members virtual and in-person primary care access for a modest monthly fee [19]. These well-known newcomers in the health space could be the key to improving access to health care going forward, eliminating barriers to travel, technological gaps, and infrastructure.

Mass General Brigham has also recently partnered with Best Buy to provide tech support and on-site care deployment, enabling more effective home patient monitoring [20]. These changes are important and will help traditional providers understand how to invest and operate to deliver value-based care based on what they do best and have opportunities to partner with others to augment remote services.

Access to health care and digital services in rural communities has historically been limited by inadequate infrastructure and poor connectivity. With the shift to digital health and telemedicine, these disparities threaten to worsen [21]. Bridging this gap requires collaboration at all levels, including community education, outreach, and availability of connected public spaces, as well as state and national investment in rural infrastructure. Advancements in telecommunications technology such as unmanned aerial vehicles and satellite connectivity (SpaceX’s Starlink) offer new solutions for remote areas [22]. Additionally, we can leverage public spaces for telehealth services, such as libraries and community centers, which already provide internet connections and private rooms. Increasing our investment in infrastructure, IT support, and cybersecurity for these spaces can address privacy and safety concerns, while training existing staff can help improve digital literacy for users of these services [23].

## Conclusion

For the first time in history, and at scale, 2025 will be characterized by digital health and human performance solutions that support consumers to fully participate in their care and drive improved health care outcomes. Physicians will be able to embrace new digital tools to deliver better care, enjoy richer patient partnerships, and enhance their health care outcomes. Health care systems that exist and are emerging will have unprecedented opportunities to deliver multiple levels of care in multiple locations, solving for needed cost, workforce, and outcome improvements.
